# A point mutation in *MC06g1112* encoding FLOWERING LOCUS T decreases the first flower node in bitter gourd (*Momordica charantia* L.)

**DOI:** 10.3389/fpls.2023.1153208

**Published:** 2023-10-10

**Authors:** Jian Zhong, Junjie Cui, Mingjun Miao, Fang Hu, Jichi Dong, Jia Liu, Chunfeng Zhong, Jiaowen Cheng, Kailin Hu

**Affiliations:** ^1^ Key Laboratory of Biology and Genetic Improvement of Horticultural Crops (South China), College of Horticulture, South China Agricultural University, Guangzhou, China; ^2^ Horticulture Research Institute, Sichuan Academy of Agricultural Sciences, Chengdu, Sichuan, China; ^3^ Department of Horticulture, Foshan University, Foshan, China; ^4^ Henry Fok School of Biology and Agricultural, Shaoguan University, Shaoguan, China

**Keywords:** bitter gourd, first flower node, quantitative trait locus, fine-mapping, *FLOWERING LOCUS T*

## Abstract

In Cucurbitaceae crops, the first flower node (FFN) is an important agronomic trait which can impact the onset of maturity, the production of female flowers, and yield. However, the gene responsible for regulating FFN in bitter gourd is unknown. Here, we used a gynoecious line (S156G) with low FFN as the female parent and a monoecious line (K8-201) with high FFN as the male parent to obtain F_1_ and F_2_ generations. Genetic analysis indicated that the low FFN trait was incompletely dominant over the high FFN trait. A major quantitative trait locus (QTL)-*Mcffn* and four minor effect QTLs-*Mcffn1.1*, *Mcffn1.2*, *Mcffn1.3*, and *Mcffn1.4* were detected by whole-genome re-sequencing-based QTL mapping in the S156G×K8-201 F_2_ population (n=234) cultivated in autumn 2019. The *Mcffn* locus was further supported by molecular marker-based QTL mapping in three S156G×K8-201 F_2_ populations planted in autumn 2019 (n=234), autumn 2020 (n=192), and spring 2022 (n=205). Then, the *Mcffn* locus was fine-mapped into a 77.98-kb physical region on pseudochromosome MC06 using a large S156G×K8-201 F_2_ population (n=2,402). *MC06g1112*, which is a homolog of *FLOWERING LOCUS T* (*FT*), was considered as the most likely *Mcffn* candidate gene according to both expression and sequence variation analyses between parental lines. A point mutation (C277T) in *MC06g1112*, which results in a P93S amino acid mutation between parental lines, may be responsible for decreasing FFN in bitter gourd. Our findings provide a helpful resource for the molecular marker-assisted selective breeding of bitter gourd.

## Introduction

The appearance of the first flower is a signal of the pivotal transition from vegetative to reproductive growth in flowering plants (Pnueli et al., 1998; Zahid et al., 2021). Both the time of first flowering and the first flower node (FFN) are useful for the evaluation of crop maturity, and are thus considered important agronomic traits in crop improvement endeavors (Yuan et al., 2008; Zhang et al., 2018; Zhang et al., 2019). In the model plant *Arabidopsis thaliana*, *FLOWERING LOCUS T* (*FT*) is an important regulator gene in determining the flowering time (Corbesier et al., 2007; Turck et al., 2008). The function of the *FT* gene has also been characterized in several Cucurbitaceae crops. In cucumber (*Cucumis sativus*), the *CsFT* gene has been reported to explain 52.3% of the phenotypic variation in flowering time, and is theorized to have been crucial to the spread of this species from its origin in the tropics to higher latitudes (Lu et al., 2014; Wang et al., 2020). The overexpression of *CsFT*, as well as *Cm-FTL1* and *Cm-FTL2* from squash (*Cucurbita maxima*) and *CmFT* from melon (*Cucumis melo*), in *Arabidopsis* promotes early flowering (Lin et al., 2007; Yang et al., 2022). Furthermore, the overexpression of *Arabidopsis*-derived *AtFT* in squash also results in early flowering (Lin et al., 2007).

Several studies have reported that other genes are also associated with the regulation of flowering time in Cucurbitaceae crops. For example, in cucumber, silencing *CsGL2-LIKE* results in delayed male flowering through inhibition of *CsFT* expression (Cai et al. (2020). Yi et al. (2020), utilizing haplotype analysis, report that *ClGA2/KS* is associated with flowering time in watermelon (*Citrullus lanatus*). The overexpression of cucumber-derived *CsTFL1b*, a homolog of *TERMINAL FLOWER 1* (*TFL1*), results in later flowering in transgenic *Arabidopsis* (Zhao et al., 2018; Cai et al., 2020). Contrarily, the overexpression of cucumber-derived *CsBCAT* (*CsBCAT2*, *CsBCAT3*, and *CsBCAT7*) and *CsMADS02* has been shown to accelerate flowering in transgenic *Arabidopsis* (Lee et al., 2019; Zhou et al., 2019). Although the function of these genes has not been universally verified, these initial reports provide clues for the further dissection of the regulation of flowering time in Cucurbitaceae crops. In addition, several quantitative trait loci (QTLs) have been reported to be associated with flowering time in cucurbits. Pan et al. (2017) and Sheng et al. (2020) identified three and two QTLs associated with flowering time in cucumber, respectively. McGregor et al. (2014) identified a major QTL associated with male flowering time in watermelon, which was later verified by Gimode et al. (2020).

Bitter gourd (*Momordica charantia*), so named because of its characteristically bitter taste, is an edible and medicinal cucurbit that has been used to treat hypertension, cancer, diabetes, infection, hyperlipidemia, and obesity (Akihisa et al., 2007; Zhang et al., 2012; Wang et al., 2017). Bitter gourd originated in Africa (Schaefer et al., 2009; Schaefer and Renner, 2010) and has become an important crop across Asia, Africa, the Caribbean, and South America, among other regions (Basch et al., 2003). In bitter gourd, low FFN or early flowering is usually considered as an important indicator of the early maturity trait. To date, genetic mapping studies have revealed at least 21 QTLs associated with female flowering time and 12 QTLs associated with male flowering time in bitter gourd (Wang and Xiang, 2013; Cui et al., 2018; Gangadhara Rao et al., 2018; Kaur et al., 2022). However, there are currently no research reports about genetic mapping of the FFN trait in bitter gourd.

Thanks to the completion of the fully sequenced and assembled bitter gourd genome (Urasaki et al., 2017; Cui et al., 2020; Matsumura et al., 2020), the mapping and cloning of genes controlling important agronomic traits has become easier. Like typical cucurbits species such as cucumber or melon, there are many types of sexual plants in bitter gourd, of which monoecy that carries both unisexual male and female flowers and gynoecy that harbors only female flower have been reported (Kole, 2020; Zhong et al., 2023). Here, we used a segregating F_2_ populations crossing from a gynoecious female parent and a monoecious male parent to elucidate the molecular mechanism of FFN regulation in bitter gourd. We first performed a whole-genome re-sequencing-based QTL mapping to rapidly identify FFN-associated genetic loci. Next, we conducted a molecular marker-based classical QTL mapping to confirm the stability of the major effected QTL in three F_2_ population cultivated three different environments, respectively. Finally, we fine-mapped the identified candidate gene. Both expression and sequence variation analyses suggest that the candidate gene *MC06g1112* regulates FFN in bitter gourd. The results of this study will be invaluable for breeding improved bigger gourds, and further our understanding of the regulation of floral timing in cucurbits.

## Materials and methods

### Plant materials

**Figure 1:**
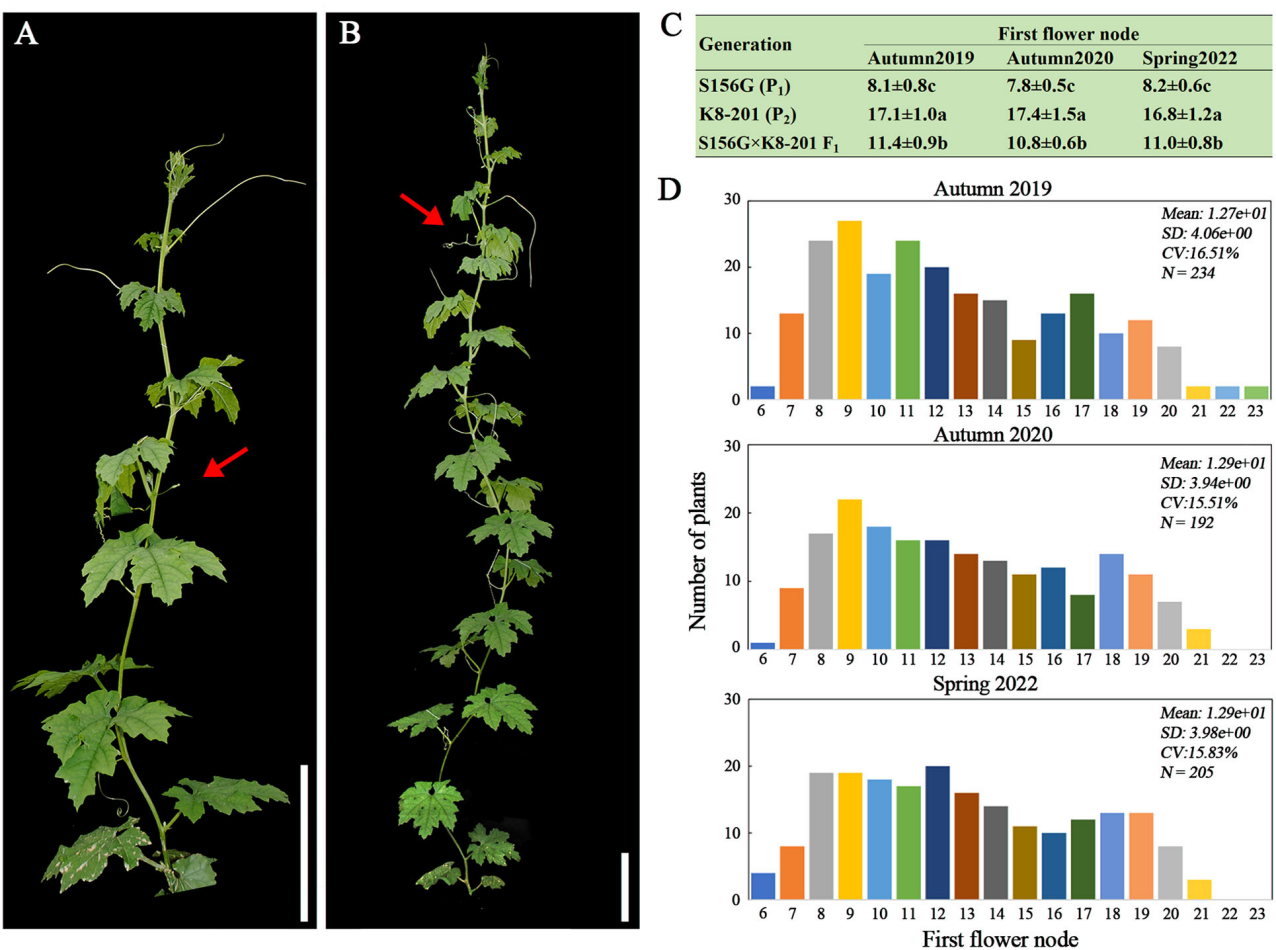
Phenotypic evaluation of the FFN trait. **(A)** Low-FFN (7-10^th^ nodes) inbred line S156G (P_1_). Bar=10 cm. **(B)** High-FFN (16-19^th^ nodes) inbred line K8-201(P_2_). Bar=10 cm. The red arrows in **(A, B)** indicate FFNs. **(C)** Average ( ± SD) FFN values of the S156G, K8-201, and S156G×K8-201 F_1_ generations recorded from autumn 2019, autumn 2020, and spring 2022. Different lowercase letters indicate statistical significance at the 0.01 level. **(D)** Phenotypic distribution of the FFN trait from the three S156G×K8-201 F_2_ populations cultivated in autumn 2019, autumn 2020, and spring 2022.

A gynoecious, low-FFN (7-10^th^ nodes) inbred line (S156G, P_1_) (**Figure 1A**) and a monecious, high-FFN (16-19^th^ nodes) inbred line (K8-201, P_2_) (**Figure 1B**) were used as the female and male parents, respectively, to construct the F_1_ generation, which was then self-crossed to generate the F_2_ population. Both of the parental lines (S156G and K8-201) had been previously whole-genome re-sequenced (Zhong et al., 2022). All plants, representing four generations (P_1_, P_2_, F_1_, and F_2_), were cultivated across three quarters (autumn 2019, autumn 2020, and spring 2022) at the experimental field of the SCAU Teaching & Research Base in Zengcheng District, Guangzhou, China (23.24N, 113.64E), under standard agronomic management. Plants from the S156G×K8-201 F_2_ population (n=234), which were cultivated in autumn 2019, were used to preliminarily map FFN-associated genetic loci by whole-genome re-sequencing-based QTL mapping. Plants from three S156G×K8-201 F_2_ populations, cultivated in autumn 2019 (n=234), autumn 2020 (n=192), and spring 2022 (n=205), were employed for molecular marker-based QTL mapping to confirm the stability of the major effected QTL. Finally, a large S156G×K8-201 F_2_ population (n=2,402) was used to fine-map the candidate region associated with FFN. The number of nodes from the node with the first alternate leaf to the node carrying the first flower was used to quantify FFN in bitter gourd.

### DNA library preparation for whole-genome re-sequencing

The CTAB method (Porebski et al., 1997) was used to isolate genomic DNA (gDNA) from young leaves, and each sample was stored at -20°C prior to analysis. gDNA isolated from the S156G×K8-201 F_2_ population (n=234) was utilized to construct DNA sequencing libraries with a KAPA-Hyper Plus Kit (KAPA Biosystems, MA, USA). Briefly, DNA was fragmented by ultrasonication to sizes of 250-350 bp, which were utilized for end-repairing and 3’ adenylation. After the adapters were ligated to the ends of these 3’-adenylated fragments, the products were purified by gel recovery. The recovered products were amplified by polymerase chain reaction (PCR) to construct the DNA sequencing libraries. The quality of the DNA sequencing libraries was evaluated using an Agilent 2100 Bioanalyzer (Agilent Technologies, CA, USA) and a Real-Time PCR (qPCR) System (Bio-Rad, CA, USA). Finally, the qualified DNA sequencing libraries were sequenced on an Illumina Nova-Seq platform (Illumina, CA, USA).

### Whole-genome re-sequencing-based QTL mapping

Quality control of raw whole-genome re-sequencing data, including removal of adapter sequences and low-quality reads, was conducted with Fastp (Chen et al., 2018). Clean reads were aligned to the Dali-11 reference genome with BMA-MEM2 (Vasimuddin et al., 2019; Cui et al., 2020), and the alignment results were evaluated with Qualimap2 (Okonechnikov et al., 2016). Both single nucleotide polymorphisms (SNPs) and insertions and deletions (InDels) were called with BCTtools (Li, 2011), and all variations were annotated with ANNOVAR (Wang et al., 2010). SNPs with a minor allele frequency <0.05 or a missing call frequency >0.1 were removed with VCFtools (Danecek et al., 2011). High quality SNPs were further used to QTL mapping using QTL package in R language. First, the multiple imputation method was used to calculate the LOD value by QTL scanning, and then the significant threshold of LOD value was obtained by 1000 permutation test. Finally, the confidence interval of the selected QTL was identified by LOD support intervals evaluation method.

### Molecular marker-based QTL mapping

The variation in SNPs and InDels between the two parental lines (S156G and K8-201) was obtained by aligning the clean re-sequencing data to the Dali-11 reference genome using SOAP2 (Li et al., 2009). Primers for SNP and InDel molecular markers within the whole-genome re-sequencing-based QTL mapping-delimited candidate region were designed with Primer3 Plus (https://www.primer3plus.com), and SNPs were converted to cleaved amplified polymorphic sequences (CAPS) or derived CAPS (dCAPS) markers. Primer sequences are listed in **Supplementary Table 1**. PCR was carried out in a 10 µL of reaction volume consisting of 0.2 µL of forward and reverse primers (10 µmol/L), 50-100 ng of DNA template, 5 µL of Green Taq Mix (Vazyme, Nanjing, China), and 3.6 µL of nuclease-free water. The PCR procedure was as follows: initial denaturation at 94 °C for 3 min; 34 cycles of denaturation at 94 °C for 15 s, annealing at 55 °C for 15 s, and extension at 72 °C for 30 s; and final extension at 72 °C for 5 min. The InDel primer-amplified PCR products were directly visualized with 6% polyacrylamide gel electrophoresis (PAGE). The CAPS- or dCAPS-amplified PCR products were first digested with corresponding restriction endonucleases (**Supplementary Table 1**) at a stationary temperature of 37 °C for 30 min, and the digested products were then visualized with 6% PAGE.

Polymorphic markers from within the molecular marker-based QTL mapping-delimited candidate region were utilized to genotype the three F_2_ populations cultivated between autumn 2019, autumn 2020, and spring 2022. Genetic distances of those polymorphic markers were calculated with JoinMap 4.0 (Van Ooijen, 2006). Based on marker genotypes and FFN phenotypes of the three F_2_ populations, FFN-associated QTL mapping was conducted with MapQTL 6.0 using the multiple QTL model (MQM mapping) procedure (Van Ooijen, 2009).

### Fine-mapping

Recombinant and non-recombinant members of the three F_2_ populations were identified using two markers flanking the candidate region identified by molecular marker-based QTL mapping. Non-recombinant plants were divided into three groups (dominant homozygote, recessive homozygote, and heterozygote) depending on whether both flanking markers were identical to S156G, K8-201, or S156G×K8-201 F_1_, respectively. During the process of fine-mapping, the average FFN values of the recessive homozygote and heterozygote groups were used as a reference to evaluate the FFN phenotype of the recombinant plant. Recombinant plants were divided into two groups: group one plants contained a recombination of the dominant homozygote and heterozygote genotypes, and group two plants contained a recombination of the recessive homozygote and heterozygote genotypes. Only group two plants were utilized for further genotyping with six newly-developed markers from within the flanked region. By using a combination of FFN phenotype data and genotype markers obtained from the group two plants, we identified a more accurate candidate region and two new flanking markers. These two new flanking markers were used to screen the S156G×K8-201 F_2_ population (n=2,402) for plants containing a recombination of the recessive homozygote and heterozygote genotypes. The selected recombinant plants were then grown in the field to evaluate their FFN phenotypes, and genotyped using nine markers from within the newly-identified flanked region. Finally, by using a combination of FFN phenotype data and genotype markers obtained from these recombinant plants, we delimited the FFN-associated fine-mapping interval.

### Expression analysis and cloning of the candidate genes

Prior to RNA extraction, tissue samples, including roots, leaves, petioles, female flowers, sarcocarps, and stems (including the 5^th^, 10^th^, 15^th^, 20^th^, and 25^th^ node [shoot tip, ST]), were collected from parental plants at the 25-leaf stage and frozen in liquid nitrogen. Three biological replicates were used for all analyses. Total RNA was extracted with an Eastep Super Total RNA Extraction kit (Promega, Shanghai, China), and first-strand cDNA was synthesized with an Eastep RT Master Mix kit (Promega, Shanghai, China), according to the manufacturer’s instructions. Quantitative real-time PCR (qRT-PCR) was performed using a TB Green Premix Ex TaqTM II kit (Takara Bio, Shiga, Japan) on a CFX384 Real-Time System (Bio-Rad, CA, USA). All primers are listed in **Supplementary Table 1**. Six categories of tissue samples, including roots, stems (15^th^ node), leaves, petioles, female flowers, and sarcocarps, were utilized to perform qRT-PCR for the genes annotated within the fine-mapped interval. The five categories of stem samples were utilized to perform qRT-PCR for the FNN-associated candidate gene. Three technical replicates were used for all assays. The relative expression level of each gene was normalized using the bitter gourd beta-actin gene (*MC02g1395*) and quantified using the delta-delta Ct method (2^-ΔΔCt^) (Livak and Schmittgen, 2001).

The primer sequences used to clone the full-length cDNA of the FNN-associated candidate gene were designed according to the gene annotation of Dali-11 reference genome (Cui et al., 2020) (**Supplementary Table 1**). PCR amplifications of cDNA collected from parental stem (15^th^ node) samples were performed with Phanta Max Super-Fidelity DNA Polymerase (Vazyme, Nanjing, China), according to the manufacturer’s instructions. The PCR products were purified and then ligated into the pMD19-T vector (Takara, Shiga, Japan). At least three positive colonies per amplicon were selected for Sanger sequencing, and the generated sequences were assembled with ContigExpress (Lu and Moriyama, 2004). Both nucleotide and amino acid sequences were aligned with ESPript 3.0 (https://espript.ibcp.fr/ESPript/cgi-bin/ESPript.cgi).

## Results

### FFN phenotypic characteristics across four generations

We evaluated the FFN phenotype of each plant across four generations, including P_1_ (S156G), P_2_ (K8-201), F_1_, and F_2_, planted respectively in autumn 2019, autumn 2020, and spring 2022. Across all three quarters, the FFN of the P_1_ (S156G) was significantly lower than that of the P_2_ (K8-201) generation, with the FFN of the P_1_ (S156G) generation ranging from the 7^th^ to the 9^th^ node (average of ~8^th^ node) and the FFN of the P_2_ (K8-201) generation ranging from the 15^th^ to the 19^th^ node (average of ~17^th^ node) (**Figures 1A-C**). The FFN of the F_1_ generation was significantly higher than the P_1_ (S156G) generation and lower than the P_2_ (K8-201) generation, ranging from the 9^th^ to the 13^th^ node (average of ~11^th^ node) (**Figure 1C**), indicating that the low FFN trait is incompletely dominant over the high FFN trait. The FFN of plants in the three F_2_ populations was highly stratified but tended toward the low FFN of the P_1_ (S156G) generation, ranging from the 6^th^ to the 23^th^ node (**Figure 1D**; **Supplementary Table 2**).

### Whole-genome re-sequencing-based QTL mapping detects FFN-associated genetic locus

**Figure 2:**
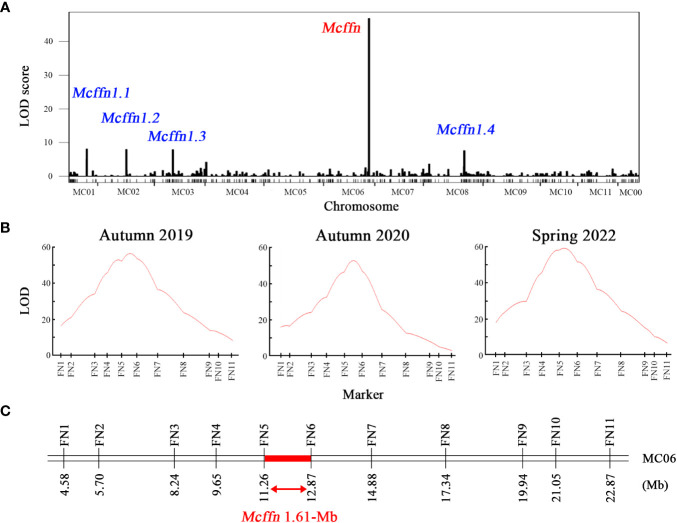
Preliminary mapping of the *Mcffn* locus. **(A)** Whole-genome re-sequencing-based QTL mapping in the S156G×K8-201 F_2_ mapping population (n=234) planted in autumn 2019. **(B)** Molecular marker-based QTL mapping in the *Mcffn* locus in three S156G×K8-201 F_2_ mapping populations planted in autumn 2019, autumn 2020, and spring 2022. **(C)** Physical map of the molecular markers used for molecular marker-based QTL mapping. The numbers under the bar correspond to the physical positions (Mb). The red bar represents the region of the *Mcffn* locus delimited by QTL analysis.

Whole-genome re-sequencing of the 234 F_2_ individuals planted in autumn 2019 resulted in the generation of 557.9 Gb of raw data, and 533.9 Gb of clean data was obtained after filtering (**Supplementary Table 3**). The clean data exhibited a Q20 of 97.3% and an average sequencing depth of 7.6×, indicating that the data was of high quality enough for subsequent bioinformatics analysis. Approximately 98.0% of the clean reads were aligned to Dali-11 reference genome, with a sample-specific genomic coverage of 88.6% (**Supplementary Table 4**). After aligning the clean reads to the Dali-11 reference genome (Cui et al., 2020), a total of 175,019 high-quality SNPs were obtained (**Supplementary Figure 1**). A QTL mapping combining FFN phenotype and SNP data from the 234 F_2_ individuals identified one ~3.77 Mb FFN-associated major effected QTL designated as the *Mcffn* locus located between 9.26 Mb and 13.03 Mb on pseudochromosome MC06 (hereafter referred to as MC06), and four minor effected QTLs named *Mcffn1.1*, *Mcffn1.2*, *Mcffn1.3*, and *Mcffn1.4* located in pseudochromosome MC01, MC02, MC03, and MC08, respectively. (**Figure 2A**, **Supplementary Table 5**).

### The *Mcffn* locus is narrowed into a 1.61-Mb interval by molecular marker-based QTL mapping

Eleven polymorphic InDel markers (FN1-FN11) were developed within the ~3.77 Mb candidate region (**Supplementary Table 1**) and used to genotype 631 F_2_ individuals cultivated in autumn 2019 (n=234), autumn 2020 (n=192), and spring 2022 (n=205). QTL mapping combining FFN phenotype and marker genotype data revealed that the 11 polymorphic InDel markers exhibited different LOD values between the three quarters: 8.50-53.81 in autumn 2019, 3.27-47.01 in autumn 2020, and 6.90-58.70 in spring 2022 (**Figure 2B**). These results suggested that all of the 11 InDel markers were linked to the FFN phenotypes. It is worth noting that the maximum LOD values of 56.73, 52.87, and 59.76, which explained 67.3%, 71.9%, and 73.9% of the variation in the FFN phenotype in the three F_2_ populations planted in autumn 2019, autumn 2020, and spring 2022, respectively, were all located between two markers, FN5 and FN6 (**Figure 2B**). Accordingly, we suggested that the *Mcffn* locus was located within a 1.61-Mb physical interval between the FN5 (11,262,463 bp) and FN6 (12,873,591 bp) markers on MC06 (**Figure 2C**).

### The *Mcffn* locus is fine-mapped into a 77.98-kb interval

Based on genotyping with the two flanking markers (FN5 and FN6), the 631 F_2_ individuals were divided into 579 non-recombinant plants and 52 recombinant plants. In order to study the relationship between *Mcffn* genotypes and FFN phenotypes, the 579 non-recombinant plants were divided into three groups: 142 dominant homozygotes, 128 recessive homozygotes, and 309 heterozygotes. The FFN phenotype was significantly different between groups across all three quarters, while within-group differences were not significant (**Table 1**), suggesting that the FFN trait is genetically, rather than environmentally, determined. To reduce the possibility of errors when categorizing plants as either dominant homozygotes or heterozygotes during the fine-mapping process, we used the FFN value of 11.6 ± 2.6 for the heterozygote genotype and 18.3 ± 2.0 for the recessive homozygote genotype (autumn 2019) as reference criteria (**Table 1**).

**Table 1:** Statistical analysis of FFN trait across three genotypes.

Genotype^a^	Autumn 2019			Autumn 2020			Spring 2020		
	No.	Mean ± SD^e^	SE^g^	No.	Mean ± SD	SE	No.	Mean ± SD	SE
RH^b^	50	18.3 ± 2.0 a^f^	0.28	38	18.4 ± 1.4 a	0.23	40	18.2 ± 1.7 a	0.20
H^c^	112	11.6 ± 2.6 b	0.24	94	12.2 ± 2.3 b	0.24	103	12.2 ± 2.2 b	0.22
DH^d^	54	8.9 ± 1.6 c	0.23	40	8.8 ± 1.5 c	0.24	48	8.5 ± 1.7 c	0.27

a Genotype determined by FN5 and FN6,

b Recessive homozygote,

c Heterozygote,

d Dominant homozygote,

e The average FFN value ± standard deviation,

f Different lowercase letters indicate significance at the 0.01 level,

g Standard error.

**Figure 3:**
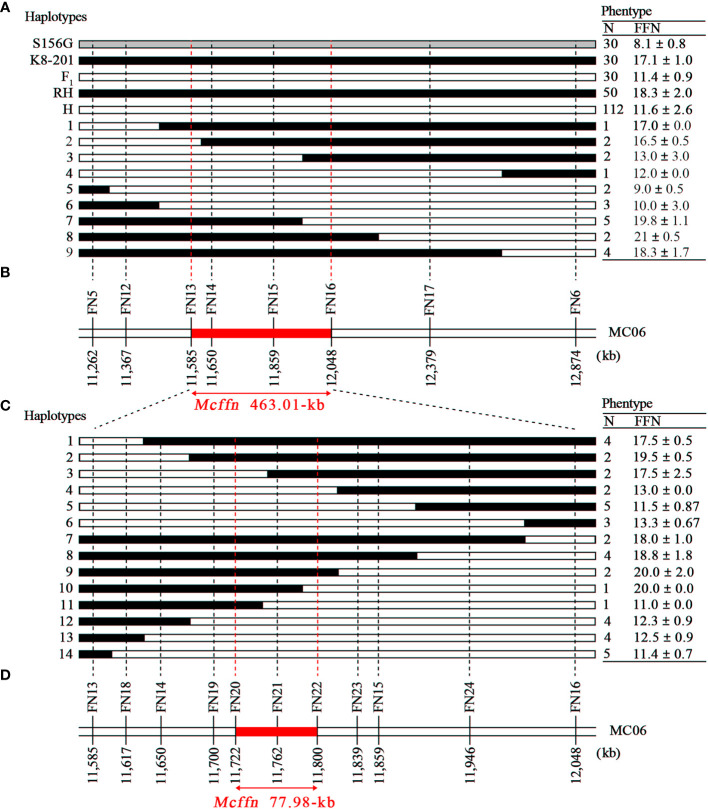
Fine-mapping of the *Mcffn* locus. **(A)** Nine haplotypes representing the 22 recombinant plants screened (with flanking markers FN5 and FN6) from the three F_2_ populations planted in autumn 2019, autumn 2020, and spring 2022. The dotted red lines indicate the boundaries of the *Mcffn* locus. RH, recessive homozygote. H, heterozygote. N, number. FFN, first flower node. **(B)** Physical map of the molecular markers used to genotype the 22 recombinant plants. The red bar represents the *Mcffn* locus. **(C)** Fourteen haplotypes representing the 41 recombinant plants screened (with flanking markers FN13 and FN16) from the large F_2_ population (n=2,402). **(D)** Physical map of the molecular markers used to genotype the 41 recombinant plants (fine-mapping).

The 52 recombinant plants were divided into two groups: group one plants (n=30) contained a recombination of the dominant homozygote and heterozygote genotypes, and group two plants (n=22) contained a recombination of the recessive homozygote and heterozygote genotypes. The group two plants were further divided into nine haplotypes using six newly-developed markers (FN12-FN17) (**Figure 3A**). By utilizing the FFN phenotype and marker genotype data of the group two plants, as well as the FFN as the reference, the *Mcffn* locus was further mapped into a 463.01-kb physical interval between the FN13 (11,585,385 bp) and FN16 (12,048,398 bp) markers on MC06 (**Figures 3A, B**).

To determine a more precise region for the *Mcffn* locus, the S156G×K8-201 F_2_ population (n=2,402) was genotyped using the two new flanking markers (FN13 and FN16). Of these, 41 plants containing a recombination of the recessive homozygote and heterozygote genotypes were obtained. Using the FN14 and FN15 markers, and seven newly-developed markers (FN18-FN24), these recombinant plants were divided into 14 haplotypes (**Figure 3C** and **Supplementary Table 1**). By utilizing the FFN phenotype and marker genotype data of the 41 recombinant plants, as well as the FFN reference criteria, the *Mcffn* locus was finally fine-mapped into a 77.98-kb physical interval between the FN20 (11,722,144 bp) and FN22 (11,800,118 bp) markers on MC06 (**Figures 3C, D**).

### Differential expression reveals *MC06g1112* as the *Mcffn* candidate gene

**Figure 4:**
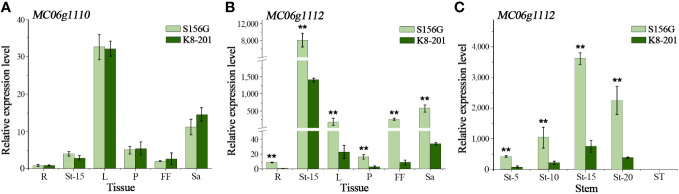
Relative expression of candidate genes. **(A)** The relative expression of *MC06g1110* in different tissues at the 25-leaf stage. **(B)** The relative expression of *MC06g1112* in different tissues at the 25-leaf stage. **(C)** The relative expression of *MC06g1112* at different stem node at the 25-leaf stage. R, root. L, leaf. P, petiole. FF, female flower. Sa, sarcocarp. St-5, stem at 5^th^ node. St-10, stem at 10^th^ node. St-15, stem at 15^th^ node. St-20, stem at 20^th^ node. ST, stem at 25^th^ node. The expression levels are presented as the mean ± SD (n=3). ** represents significance at the 0.01 level (Student’s t test).

By examining the annotation of the Dali-11 reference genome (Cui et al., 2020), we identified four annotated genes (*MC06g1110*, *MC06g1111*, *MC06g1112*, and *MC06g_new0263*) within the 77.98-kb fine-mapping interval. We considered *MC06g1111* a pseudogene because its predicted cDNA is only a 72-bp short nucleotide fragment lacking a complete gene structure and because no transcripts were detected in any sampled tissues (Cui et al., 2020). Only minimal (Cq>35) expression was detected for *MC06g_new0263* across tissues in both parental lines, and no significant differences in the relative expression of *MC06g1110* were detected across tissues between parental lines (**Figure 4A**). However, *MC06g1112* exhibited significantly different relative expression across tissues between parental lines (**Figure 4B**). Furthermore, *MC06g1112* exhibited different relative expression across the five different stem categories, increasing from the 5^th^ to the 15^th^ node, and decreasing from the 15^th^ to the 25^th^ node, with almost no expression at the ST (**Figure 4C**). Additionally, *MC06g1112* exhibited significantly higher expression in the stems of P_1_ (S156G) plants than in the stems of P_2_ (K8-201) plants at all nodes from the 5^th^ to the 20^th^ (**Figure 4C**). Accordingly, we proposed that *MC06g1112* was the FFN-associated *Mcffn* candidate gene.

### A point mutation of *MC06g1112* may decrease the FFN

**Figure 5:**
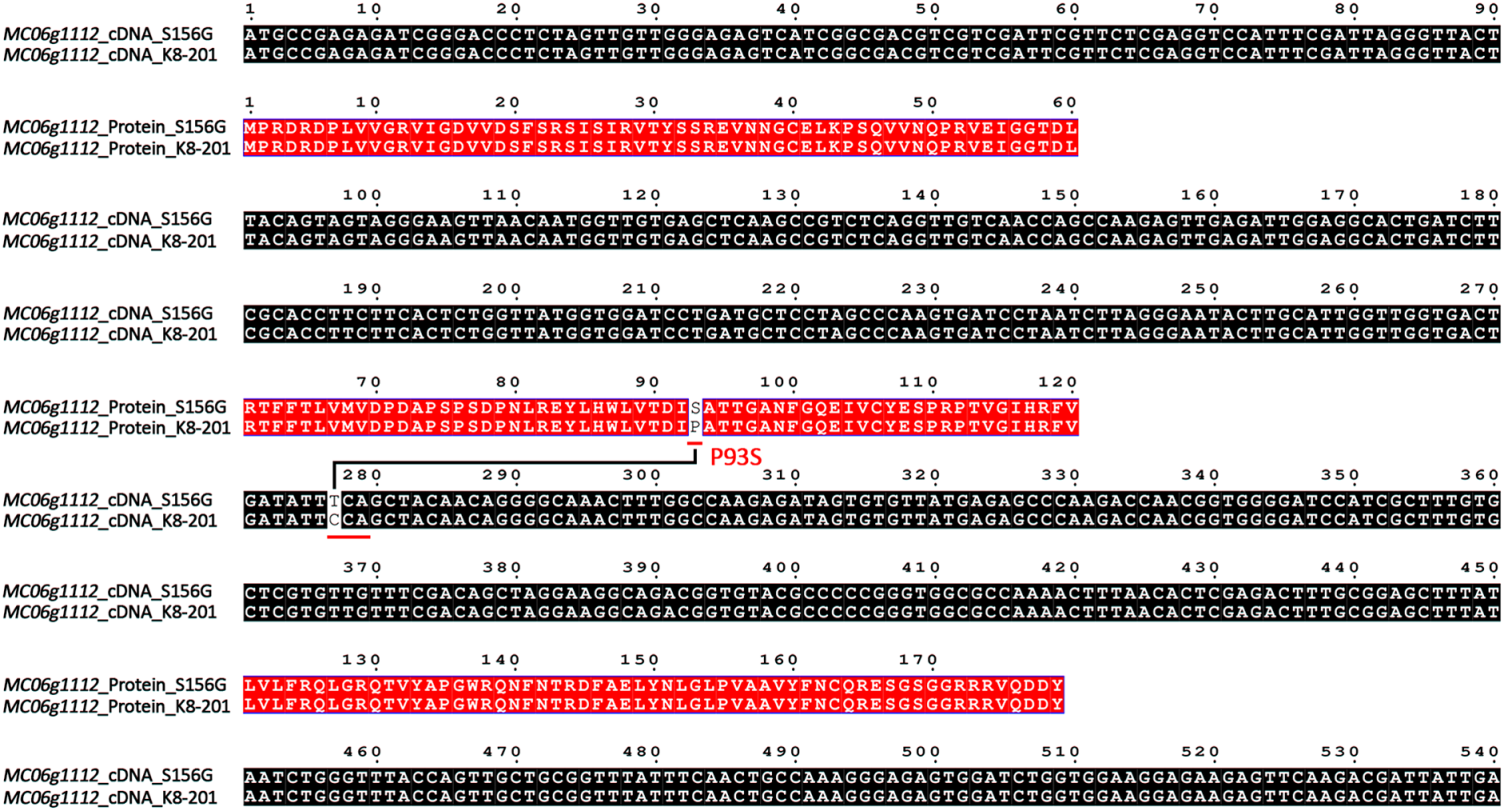
Alignment of full-length cDNA and amino acid sequences of *MC06g1112* between S156G and K8-201. Black boxes represent cDNA sequences and red boxes represent amino acid sequences.

By comparing the genomic sequences of the parental lines, we identified seven single-nucleotide variations (SNV-1~SNV-7) within the 77.98-kb fine-mapping interval (**Supplementary Table 6**). Of these, only SNV-2 (11,775,926 bp) was located within the *MC06g1112* coding region, while the other six SNVs were located in the intergenic spacer region (**Supplementary Table 6**). The full-length *MC06g1112* cDNA sequences of parental lines were cloned and compared, as a result, *MC06g1112* gene consisted of 540 base pairs, which is a homolog of the *FT* gene encoding a phosphatidylethanolamine-binding protein (PEBP), and therefore was called *McFT*; additionally, we identified a point mutation (C>T) located 277 bp away from the *MC06g1112* start codon, which leaded proline (P) of K8-201 to serine (S) of S156G (hereinafter referred to as P93S) (**Figure 5**).

To further verify the association of SNV-2 (C277T) and FFN, we designed a dCPAS marker to target SNV-2 by introducing a mismatched base (C) at the end of forward primer to create a *Msp* I restriction enzyme site, which can theoretically produce a 184-bp single fragment with the DNA template of K8-201, a 205-bp single fragment with the DNA template of S156G, and double fragments of 184-bp and 205-bp with the DNA template of S156G×K8-201 F_1_ generation (**Supplementary Figure 2**). Actually, however, the results of marker genotyping showed that both S156G and S156G×K8-201 F_1_ generation displayed double fragments of 184-bp and 205-bp, and K8-201 displayed a single 184-bp fragment; in 234 S156G×K8-201 F_2_ individuals (autumn 2019), all dominant homozygous and heterozygous plants exhibited double fragments of 184-bp and 205-bp, and all recessive homozygous plants exhibited a single 184-bp fragment (**Supplementary Figure 3**). Therefore, we conducted Sanger sequencing targeting SNV-2, which indicated that the SNV-2 locus in both DNA and cDNA of K8-201 were recessive homozygous genotype (C), in cDNA of S156G was dominant homozygous genotype (T), while in DNA of S156G was heterozygous genotype (C/T) (**Supplementary Figure 4**), which implied that the region where SNV-2 is located might have two or multiple copies on the bitter gourd genome.

**Figure 6:**
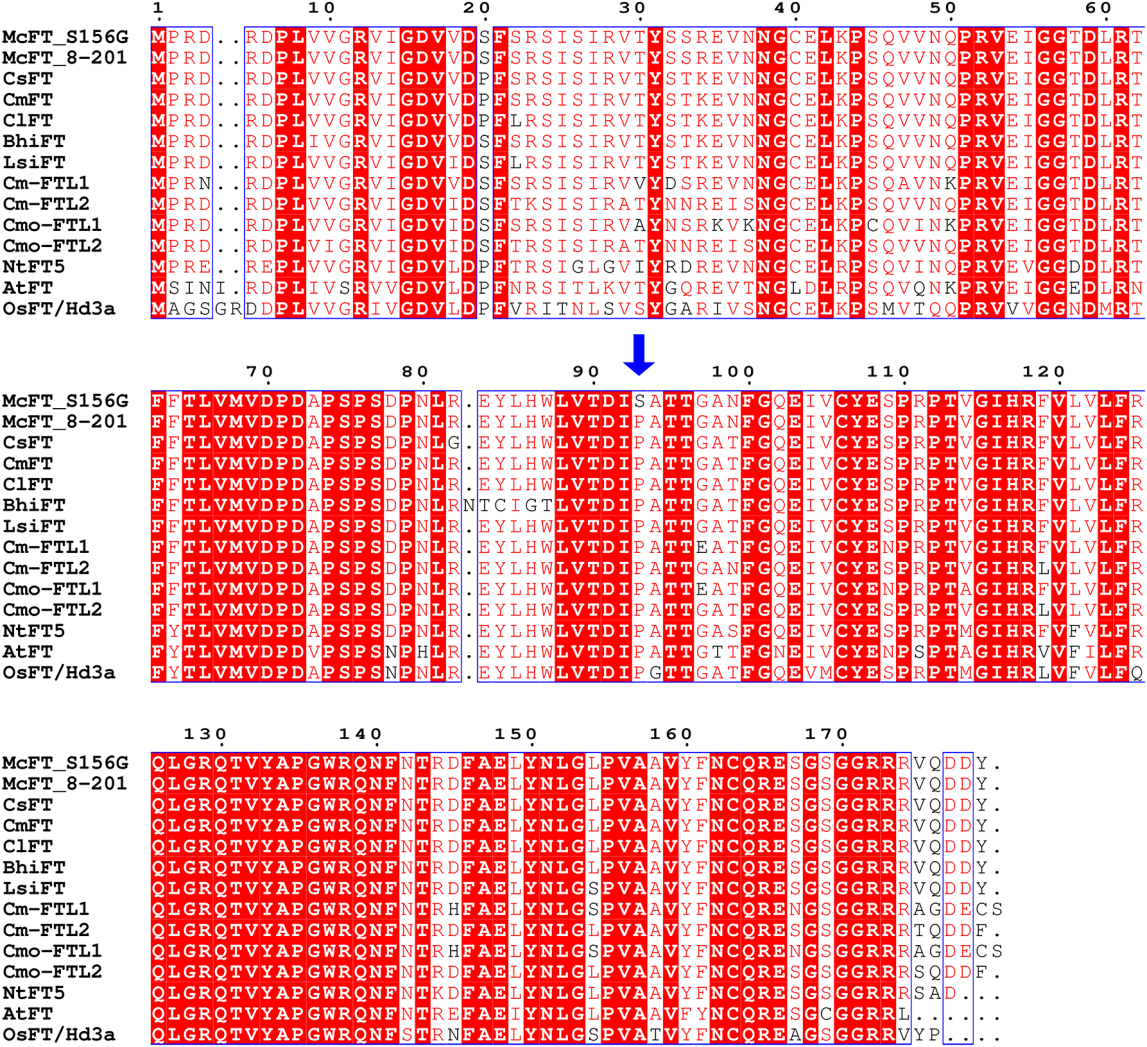
Alignment of amino acid sequences of FT proteins across different species of flowering plants. McFT, *Momordica charantia*; CsFT, *Cucumis sativus*; CmFT, *Cucumis melo*; ClFT, *Citrullus lanatus*; BhiFT, *Benincasa hispida*; LsiFT, *Lagenaria siceraria*; NtFT5, *Nicotiana tabacum*; OsFT/Hd3a, *Oryza sativa*; AtFT, *Arabidopsis thaliana;* Cm-FTL1 and Cm-FTL2, *Cucurbita maxima*; Cmo-FTL1 and Cmo-FTL2, *Cucurbita moschata*. The blue arrow indicates the P93S amino acid mutation. The source of each FT protein sequence is listed in **Supplementary Table 7**.

The McFT proteins of the two parental lines were compared with previously-characterized FT from *C. sativus* (CsFT), *C. melo* (CmFT), *C. lanatus* (ClFT), *Benincasa hispida* (BhiFT), *Lagenaria siceraria* (LsiFT), *Cucurbita maxima* (Cm-FTL1 and Cm-FTL2), *Cucurbita moschata* (Cmo-FTL1 and Cmo-FTL2), *Nicotiana tabacum* (NtFT), *Oryza sativa* (OsFT/Hd3a), and *Arabidopsis thaliana* (AtFT). Sequence alignment and phylogenetic analysis indicated that the McFT proteins from S156G and K8-201 were highly homologous with these previously-characterized FTs, especially FTs of cucurbits species, sharing between 73.63 and 97.21% sequence identity (**Figure 6** and **Supplementary Figure 5**). Additionally, P93 was a strictly-conserved amino acid across all of the examined species, with only one mutant S93 identified in S156G (**Figure 6**). We speculated that the P93S mutation might be responsible for the decreased FFN exhibited by S156G, since the FFN of S156G was significantly lower than that of K8-201.

## Discussion

The onset of flowering, which signals the transition from vegetative to reproduction growth, is a particularly important agronomic trait in Cucurbitaceae crops, as this trait can influence the onset of maturity, the production of female flowers, and yield (Lu et al., 2014; Zhao et al., 2018; Wen et al., 2019). Previous research on the timing of flowering in bitter gourd has primarily focused on the time of onset of flowering, either female or male, from sowing, and has led to the identification of flowering-associated QTLs through genetic mapping (Wang and Xiang, 2013; Cui et al., 2018; Gangadhara Rao et al., 2018; Kaur et al., 2022). However, the gene responsible for regulating flowering time in bitter gourd remained unidentified.

As one of the model plants for research on sex differentiation, Cucurbitaceae species harbor all three basic types of flower sexes, namely female, male, and hermaphroditic flowers (Dellaporta and Calderon-Urrea, 1993; Schaefer and Renner, 2011). All these three basic types carry both pistil and stamen primordia at early development stage of flower bud, and the formation of female and male flowers are resulted by the arrest of stamen and pistil development, respectively (Bai et al., 2004). Previous studies have revealed that the “arrest” processes are genetically controlled, such as the loss of function of *CmWIP1*, *CsWIP1*, and *ClWIP1* lead to gynoecious lines in melon, cucumber, and watermelon, respectively (Martin et al., 2009; Hu et al., 2017; Zhang et al., 2020). Also, our previous works using S156G×K8-201 F_2_ population have confirmed that the gene locus responsible for gynoecy in bitter gourd is located at the end of MC01 (Zhong et al., 2023). In addition, our results of phenotype investigation showed that there was no direct relationship between gynoecy and FFN in the three S156G×K8-201 F_2_ populations (**Supplementary Table 2**). Hence, we speculate that gynoecy and FFN are independently inherited in bitter gourd.

Here, we used FFN as a proxy for flowering time in bitter gourd and detected a main effect QTL (*Mcffn*) associated with FFN via whole-genome re-sequencing-based QTL mapping (**Figure 2A**). Then molecular marker-based QTL mapping indicated that *Mcffn* could explain 67.3-73.9% of the variation in the FFN phenotype (**Figure 2B**), which is higher than the explanatory power reported for QTLs related to either female or male flowering time in bitter gourd (Wang and Xiang, 2013; Cui et al., 2018; Gangadhara Rao et al., 2018; Kaur et al., 2022). Furthermore, the consecutive variation in FFN exhibited by the segregating F_2_ populations (**Figure 1D**) implies that there may be multiple QTLs associated with flowering time in bitter gourd, which is consistent with our QTL mapping results (**Figure 2A**). Based on fine-mapping, gene expression, and sequence comparison analyses, the *MC06g1112* (*McFT*) gene was identified as the most likely FFN-associated *Mcffn* candidate gene (**Figures 3**–**5**). In cucumber, *CsFT* has been found to explain 52.3% of the variation in flower time, and two large structural variations upstream of *CsFT* are associated with earlier flowering (Lu et al., 2014; Zhao et al., 2018; Gimode et al., 2020). Previous comparative genome analyses have shown that most of genomic sequences of pseudochromosome MC06 of bitter gourd, including the genomic fragment where *McFT* is located, are mapped to the chromosome 1 of cucumber (Cui et al., 2020). It is worth mentioning that *CsFT* is just in this collinear genomic interval (Lu et al., 2014), which may imply that bitter gourd and cucumber evolved from the same ancestor and the molecular mechanism regulating FFN or flowering time is highly similar in bitter gourd and cucumber. In squash, the ectopic expression of *Arabidopsis*-derived *AtFT*, which is responsive to inductive short-day (SD) photoperiods, has highly effective in mediating floral induction under long-day (LD) treatment (Lin et al., 2007). These evidences indicate that *FT* genes of cucurbit species may be conserved, and thus its development and application are beneficial to early maturity breeding for cucurbit crops.

Several previous studies have confirmed that the *FT* gene is the downstream target of many transcription factors (TFs) associated with flowering time, such as *CONSTANS* (*CO*), *PHYTOCHROME INTERACTING FACTOR4* (*PIF4*), *FLOWERING LOCUS C* (*FLC*), and *PHYTOCHROME AND FLOWERING TIME 1* (*PFT1*), among others (Putterill et al., 1995; Cerdán and Chory, 2003; Crevillén and Dean, 2011; Kumar et al., 2012), and hence plays a vital role in regulating flowering time across diverse flowering plants, including *Arabidopsis*, rice (*O. sativa*), and winter oilseed rape (*Brassica napus*), among others (Komiya et al., 2008; Ho and Weigel, 2014; Vollrath et al., 2021). In *Arabidopsis*, the *FT* mRNA is expressed in the vasculature of cotyledons and leaves while the FT protein interacts with a bZIP TF (FD) in the shoot apex to promote floral transition and initiate floral development (Abe et al., 2005; Wigge et al., 2005). Lin et al. (2007), using squash as a model system, report that the FT protein is translocated long distances through the phloem to the shoot apical meristem, where it induces flowering. Because of this, FT is generally considered a long-distance signal, or a leaf-to-apex communicator, for the induction of flowering (Corbesier et al., 2007; Lin et al., 2007). In this study, we detected almost no expression of *McFT* in STs of both parental lines (**Figure 4C**), suggesting that the functional mechanism of *McFT* in bitter gourd may be similar with previously-reported squash (Lin et al., 2007). In addition, previous studies have focused on *FT* expression in cucumber mainly using leaf tissues (Lu et al., 2014; Wang et al., 2020; Yang et al., 2022). Unlike these previous studies, we examined *FT* expression in several bitter gourd tissues and found that this gene was expressed in all tissues (with the exception of STs), and the expression was particularly high in stem tissues (**Figure 4B**). Our results suggest that the stem tissue may have the greatest impact on flowering time, although the precise regulatory mechanism underlying *McFT* expression requires further study.

In general, FT is a highly conserved protein which is robust to a wide range of mutations and plays a similar functional role in many species (Lin et al., 2007; Ho and Weigel, 2014; Putterill and Varkonyi-Gasic, 2016). However, Ho and Weigel (2014) reported that the P93 mutation of the FT protein may alter flowering time in *Arabidopsis*, with the P93A and P93T mutations resulting in early flowering and the P93H mutation resulting in delayed flowering. In the present study, we displayed the conservatism of P93 of FT protein across cucurbit species and some other flowering plant species, and identified a P93S amino acid mutation of the McFT protein in bitter gourd (**Figure 6**). We speculate that the P93S mutation may be responsible for the decreased FFN exhibited by S156G (**Table 1**). Furthermore, the results of genotypes and Sanger sequencing targeting SNV-2 (C277T) suggested that the region where SNV-2 is located might have two or multiple copies on the bitter gourd genome (**Supplementary Figure 3 and 4**). The genome replication events may still be the cause of the change of FFN, such as *CsACS1G*, which is a copy of *CsACS1* and leads to gynoecy in cucumber (Mibus and Tatlioglu, 2004; Li et al., 2020). Overall, our results suggest that the *FT* genes may be highly conserved across cucurbits, and thus they should be considered targets for the molecular breeding of early-maturing Cucurbitaceae crops.

## Conclusions

FFN of bitter gourd is regulated by a major effect QTL named *Mcffn*, with the low FFN is incompletely dominant over the high FFN. The *Mcffn* locus was fine-mapped into a 77.98-kb physical region on MC06. *MC06g1112*, a homolog of *FT*, was considered as the most likely *Mcffn* candidate gene according to expression and sequence variation analyses. A point mutation (C277T) in *MC06g1112*, which results in a P93S amino acid mutation between parental lines, may be responsible for decreasing FFN in bitter gourd.

## Data availability statement

The data presented in the study are deposited in the Genome Sequence Archive in National Genomics Data Center, China National Center for Bioinformation/Beijing Institute of Genomics, Chinese Academy of Sciences repository, accession number CRA009976.

## Author contributions

KH and JWC conceived and designed the research. JZ performed most of the experiments and wrote the manuscript. JL, CFZ and MJM performed statistical analysis. JJC, FH and JCD provided helpful discussions. All authors contributed to the article and approved the submitted version.
