# Supplementary material for: A point mutation in MC06g1112 encoding FLOWERING LOCUS T decreases the first flower node in bitter gourd (Momordica charantia L.)
Source: Front Plant Sci. 2023 Oct 10;14:1153208. doi: 10.3389/fpls.2023.1153208 (PMC10595031; doi:10.3389/fpls.2023.1153208)
Supplement: Supplementary file 1 [file DataSheet_1.docx]

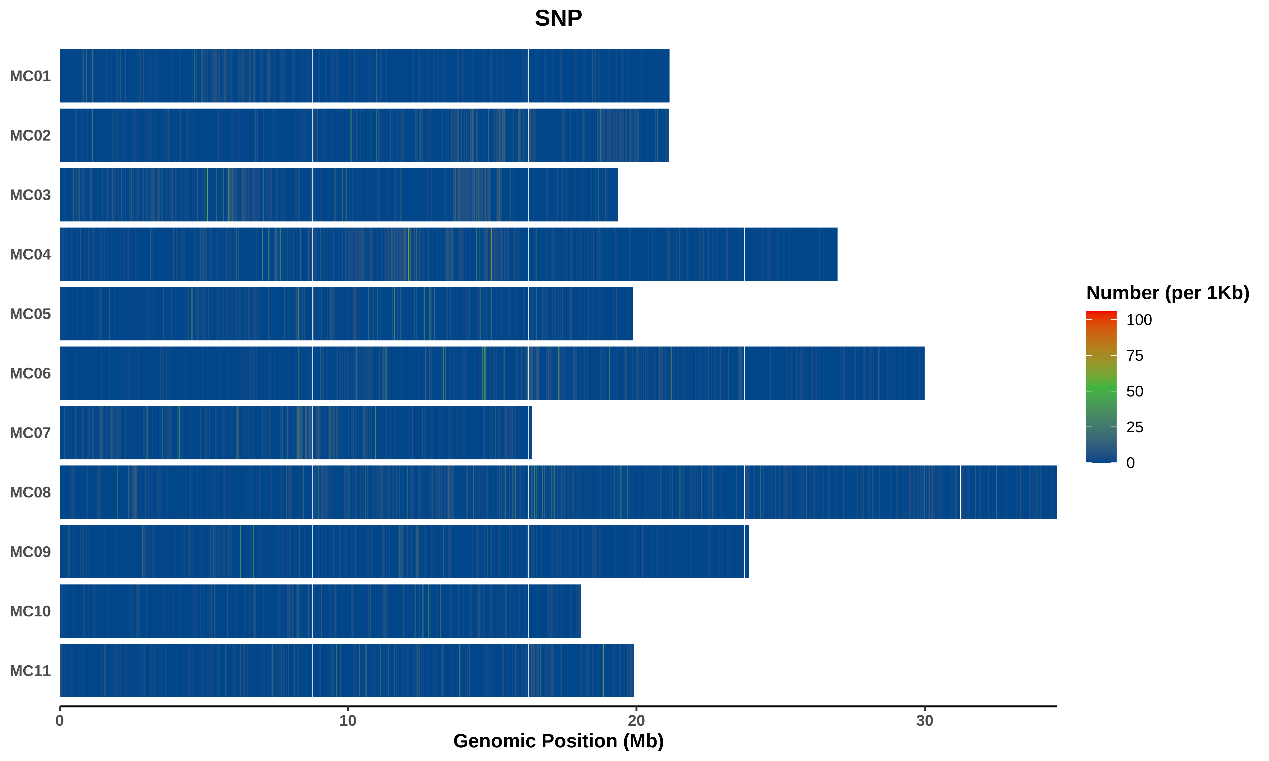


**Supplementary figure 1** Distribution map of the filtered SNPs used for QTL mapping


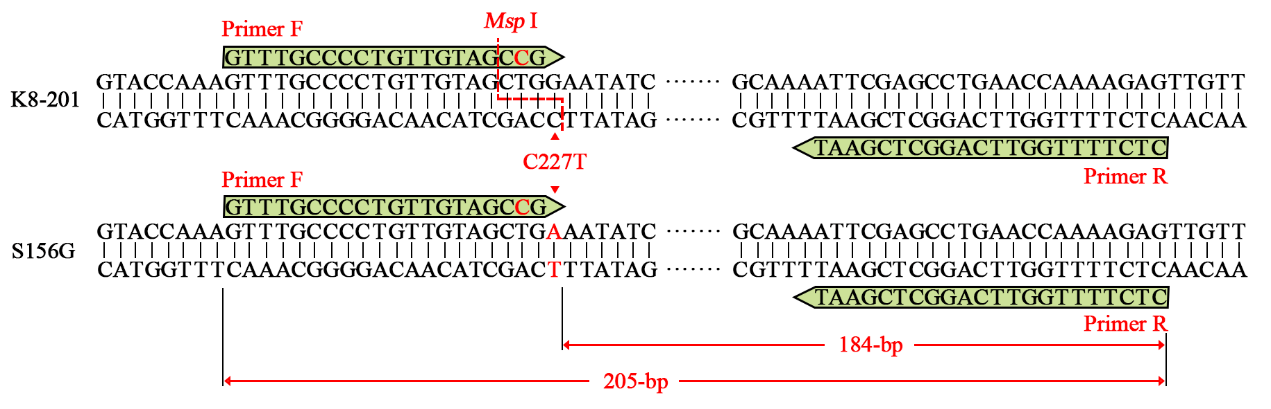


**Supplementary figure 2** The development diagram of dCAPS marker targeting SNV-2 (C227T).

The red letter, C, is a mismatched base introduced by forward primer.


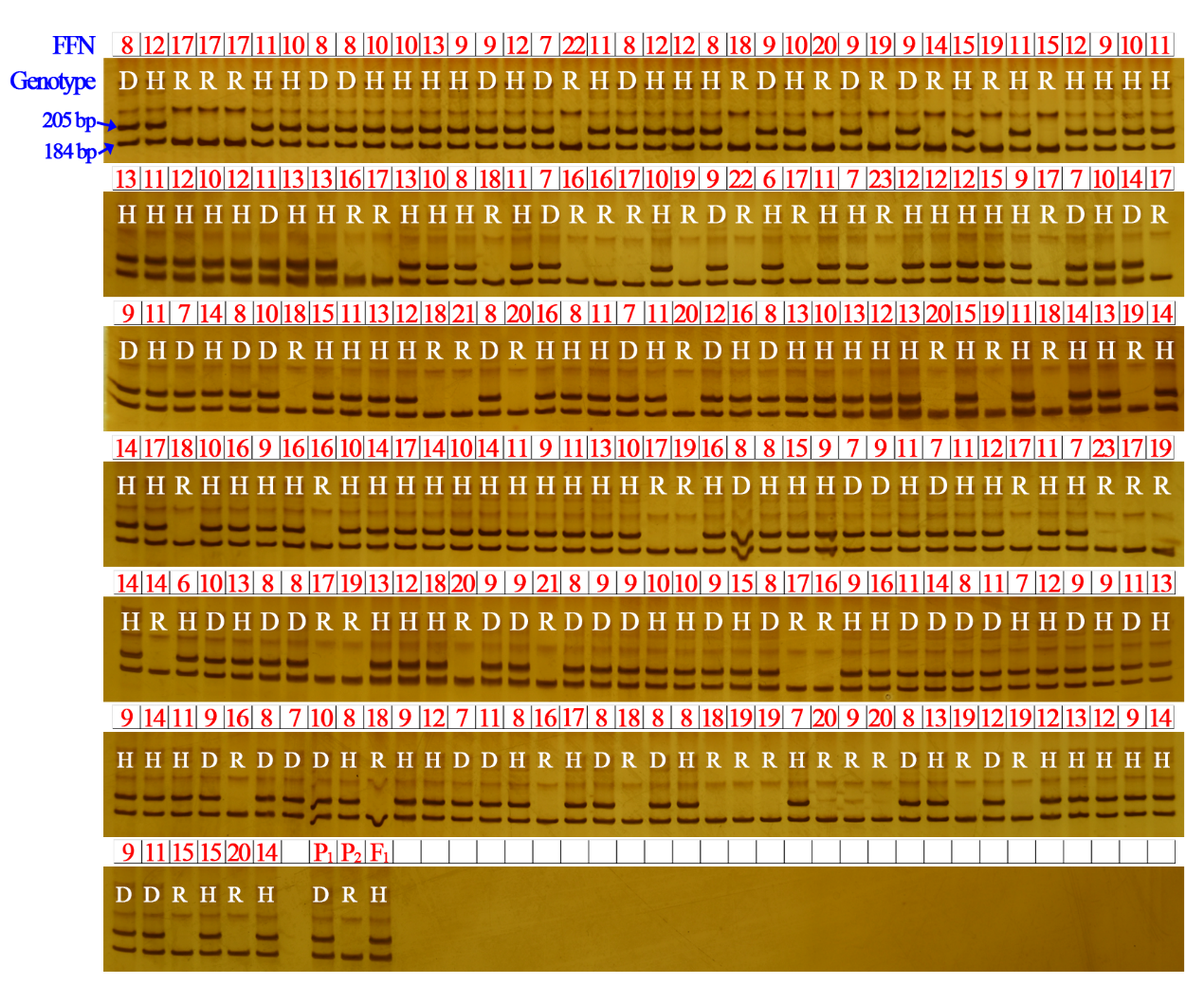


**Supplementary figure 3** Genotypes of the dCAPS marker targeting SNV-2 (C227T) in 234 S156G×K8-201 F_2_ individuals planted in autumn 2019.

The red numbers indicate first flower node (FFN). The white letters, D, H, and R, represent respectively dominant homozygous, heterozygous, and recessive homozygous genotypes determined by FN21 marker which is located in the *Mcffn* fine-mapping interval and only 14-kb away from SNV-2.


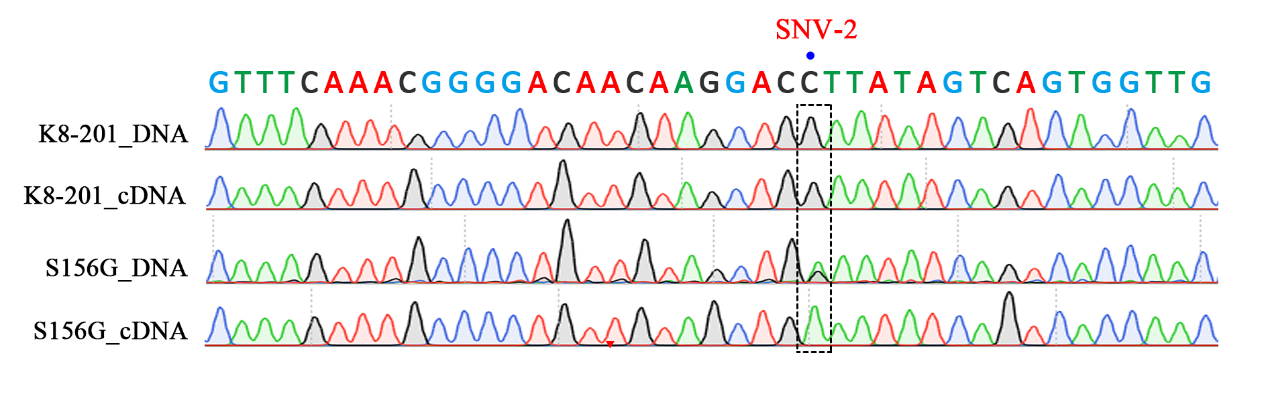


**Supplementary figure 4** Peak figures of Sanger sequencing targeting SNV-2 (C227T) with DNA and cDNA templates of S156G and K8-201.


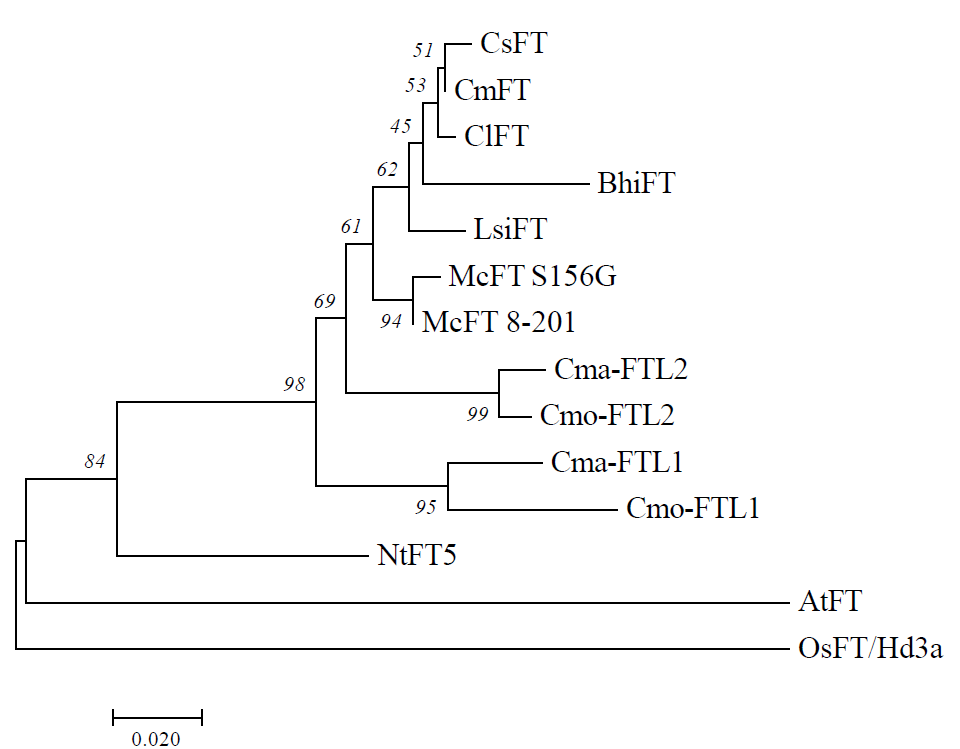


**Supplementary figure 5** Phylogenetic analysis of FTs from different species of flowering plants.

McFT, *Momordica charantia*. CsFT, *Cucumis sativus*. CmFT, *Cucumis melo*. ClFT, *Citrullus lanatus*. BhiFT, *Benincasa hispida*. LsiFT, *Lagenaria siceraria*. NtFT5, *Nicotiana tabacum*. OsFT/Hd3a, *Oryza sativa*. AtFT, *Arabidopsis thaliana*. Cm-FTL1 and Cm-FTL2, *Cucurbita maxima*. Cmo-FTL1 and Cmo-FTL2, *Cucurbita moschata*.
